# An Overview of the Obese-Asthma Phenotype in Children

**DOI:** 10.3390/ijerph19020636

**Published:** 2022-01-06

**Authors:** Valentina Fainardi, Lucrezia Passadore, Marialuisa Labate, Giovanna Pisi, Susanna Esposito

**Affiliations:** Pediatric Clinic, Department of Medicine and Surgery, University of Parma, 43126 Parma, Italy; valentina.fainardi@unipr.it (V.F.); lucre.passadore@gmail.com (L.P.); labate202@gmail.com (M.L.); gpisi@ao.pr.it (G.P.)

**Keywords:** asthma, children, gastroesophageal reflux, obesity, OSA, overweight

## Abstract

Asthma is the most common chronic disease in childhood. Overweight and obesity are included among the comorbidities considered in patients with difficult-to-treat asthma, suggesting a specific phenotype of the disease. Therefore, the constant increase in obesity prevalence in children and adolescents raises concerns about the parallel increase of obesity-associated asthma. The possible correlation between obesity and asthma has been investigated over the last decade by different authors, who suggest a complex multifactorial relationship. Although the particular non-eosinophilic endotype of obesity-related asthma supports the concept that high body weight precedes asthma development, there is ongoing debate about the direct causality of these two entities. A number of mechanisms may be involved in asthma in combination with obesity disease in children, including reduced physical activity, abnormal ventilation, chronic systemic inflammation, hormonal influences, genetics and additional comorbidities, such as gastroesophageal reflux and dysfunctional breathing. The identification of the obesity-related asthma phenotype is crucial to initiate specific therapeutic management. Besides the cornerstones of asthma treatment, lifestyle should be optimized, with interventions aiming to promote physical exercise, healthy diet, and comorbidities. Future studies should clarify the exact association between asthma and obesity and the mechanisms underlying the pathogenesis of these two related conditions with the aim to define personalized therapeutic strategies for asthma management in this population.

## 1. Introduction

Asthma is the most common chronic disease in childhood. Its prevalence ranges between 1 and 18%, depending on geographical area and is slightly increasing worldwide [1]. The disease is characterized by chronic airway inflammatory (usually eosinophilic), variable airflow limitation, and symptoms such as wheeze, shortness of breath, chest tightness and cough [2]. Asthma is a heterogeneous disease with different phenotypes (clinical presentation, natural history, response to treatment) and endotypes (underlying pathophysiological and/or molecular mechanisms) [3], but the most common variant is characterized by atopy, eosinophilic inflammation and T helper 2 cell-mediated allergic sensitization, known as T2-hi asthma [4].

Low doses of inhaled corticosteroids (ICS) are usually successful in achieving good symptom control in the majority of children with asthma. However, approximately 5% of all asthmatic children aged 6 years and older [5] remain symptomatic, have frequent exacerbations and/or persistent airflow obstruction despite maximal prescribed therapy. This subgroup of patients is currently defined as having “problematic severe asthma” [5]. Problematic severe asthma includes “difficult-to-treat asthma” due to modifiable underlying factors and true “severe, therapy-resistant asthma” (STRA) who have persistent symptoms despite optimization of the basics of asthma management [6,7].

The modifiable factors can be environmental factors such as persistent exposure to tobacco smoke, or to aero-allergens that the child is sensitised to or there may be subject-related factors contributing to poor control, such as poor inhaler technique, poor adherence to treatment and the presence of other comorbidities like rhinosinusitis, gastroesophageal reflux, food allergies, breathing pattern disorders, psychosocial issues or obesity [8].

The association of difficult-to-treat asthma with overweight and obesity suggests a specific phenotype of the disease. Therefore, the constant increase of obesity prevalence in children and adolescents raises concerns about the parallel increase of obesity-associated asthma [9]. The possible correlation between obesity and asthma has been investigated over the last decade by different authors who suggest a complex multi factorial relationship [10]. Although the particular non-eosinophilic endotype of obesity-related asthma supports the concept that high body weight precedes asthma development, there is ongoing debate about the direct causality of these two entities [11].

Many mechanisms can be involved in asthma combined with obesity disease in children including reduced physical activity, abnormal ventilation, chronic systemic inflammation, hormonal influences, genetics and additional comorbidities such as gastroesophageal reflux and dysfunctional breathing [12].

The purpose of this review is to summarize the evidence on obese asthmatic children by considering the different endotypes and phenotypes and suggesting the best educational and therapeutic approach.

## 2. Which Came First, the Chicken or the Egg?

The relationship between asthma and obesity is still a matter of debate. The prevalence of asthma and overweight has increased simultaneously in the recent decades in the paediatric population [13]. Consequently, several epidemiological studies have been conducted in order to explore the link between these two disorders. Obesity and asthma are likely related [14], but the nature of the association is not clear, and the potential causal relationship is unknown [15,16].

Numerous studies [17,18,19,20] demonstrated that in children overweight or obesity are risk factors for asthma development. Egan et al. [16] examined six prospective cohort studies and reported that compared to normal weigh children, children with overweight or obesity have a 50% increased risk of physician-diagnosed asthma. These results were supported by the meta-analysis by Chen et al. [21], who evidenced that obese children have a double risk of developing asthma with a risk proportional to body mass index (BMI) values, particularly in boys.

Overall, obesity increases the risk of asthma in all age groups across the paediatric age [13,17] but Lang et al. [22] demonstrated that the age group with the highest risk of developing obesity-related asthma was the prepubertal school-aged group (7–11 years) without allergic rhinitis. Lang et al. suggested that the onset of asthma would be driven by both duration and severity of overweight and hypothesized that the years before the onset of puberty could represent a particularly high-risk moment for obesity-related asthma, particularly in girls. This risk becomes higher in boys after 12 years old, suggesting a role of gender in obesity-related paediatric asthma.

In adults, obesity has been associated with severe asthma and exacerbations [23,24]. Barros et al. [23] conducted a cross-sectional study in Brazil, including 508 adults, and demonstrated that obese asthmatics had the highest rate of hospitalization and emergency room accesses, suggesting a poor control of the disease. Another Brazilian cross-sectional study [24] showed that obese individuals with asthma had a poorer quality of life and more frequent asthma exacerbations requiring oral corticosteroids. A 3-year cohort study demonstrated that patients with more exacerbations had increased BMI values, higher levels of systemic inflammatory markers, including blood neutrophil numbers and blood interleukin (IL)-6 levels, and higher levels of metabolic dysfunction indicators such as history of diabetes mellitus and hypertension [25]. Indicators of type-2 inflammation such as eosinophils in blood and sputum or fractional exhaled nitric oxide levels did not influence this phenotype [25]. Obese asthmatics with elevated levels of IL-6 may have an impaired natural killer (NK) cell function and lower gene expression signals for CD8 cytotoxic T cells [26], resulting in an increased susceptibility to viral illnesses and therefore a higher rate of exacerbation.

In children, a recent study of 995 subjects (17% obese) found no association between obesity and the severity of exacerbation, which is expressed as length of hospitalization and admission to an intensive care unit [27]. Despite the high prevalence of obesity in some cohort of children with severe asthma, in a retrospective cohort including data from six European electronic healthcare databases obesity was not considered as risk factor for exacerbation [28]. Orriëns et al. [29] hypothesized that overweight and obese children with asthma could have higher odds of intentional non-adherence to ICS, potentially resulting in a greater asthmatic exacerbation rate. The authors included 566 asthmatic children in their analyses, aged 4–13 years, who were undergoing maintenance therapy with ICS and evidenced that the proportion of intentional non-adherents was higher in in children with moderate-to-severe asthma with excess weight (75.8% vs. 65.0% in children with normal weight).

Other evidence supports the hypothesis that asthma may contribute to the increase in obesity onset. In the period 2002–2003 more than 5000 children were enrolled in California (US) and followed-up for 10 years [10]. This longitudinal study observed that normal weight children with asthma had a higher risk of developing obesity compared to non-asthmatic children. Chen et al. came to the same conclusion and found a further higher risk in children with asthma not controlled by rescue therapy [30].

Further studies [19,31,32] supported the hypothesis of a bidirectional association between asthma and obesity in children and adolescents. Contreras et al. [19] performed an analysis of 16 European cohorts which had followed 21.130 children from the age of 3–4 years up to 8 years of age. They found that persistent wheezing and early onset asthma were associated with a higher risk of developing obesity. Moreover, a meta-analysis [31] conducted in 2020 concluded that during childhood and adolescence there is a bidirectional association between obesity and asthma. The analysis included nine high-quality studies with six reporting a higher risk of physician-diagnosed asthma in obese children and adolescents (this risk was particularly evident in boys) and three a higher risk of obesity in children with asthma. In order to study the link between asthma and obesity, Green et al. [32] analysed the data from the Early Childhood Longitudinal Study-Kindergarten Cohort (ECLS-K), a longitudinal study that followed a cohort of children from kindergarten to middle school. The authors evidenced that asthma onset could be related to weight gain over time. However, the onset of overweight or obesity was not related to a subsequent onset of asthma, suggesting that weight gain over time, and not the initial condition of being overweight or obese, would be responsible for driving the onset of asthma. Table 1 summarizes studies supporting the higher risk of overweight and obese subjects of developing asthma.

## 3. Aetiopathogenesis of the Bidirectional Relationship between Obesity and Asthma

**Figure 1:**
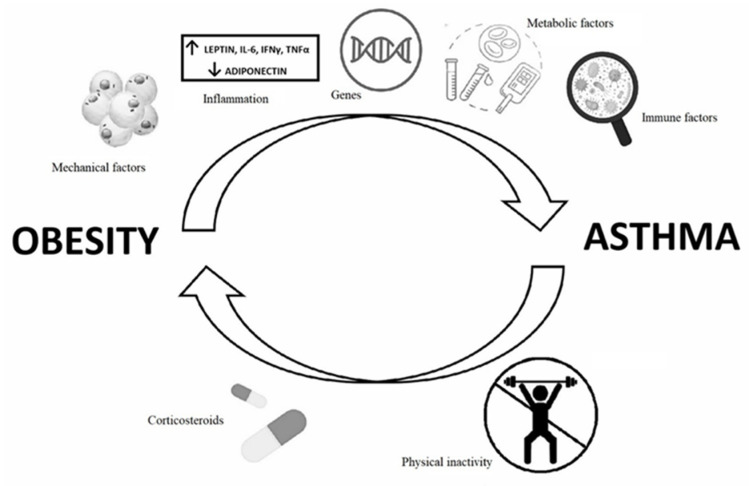
Proposed mechanisms of the bidirectional relationship between obesity and asthma.

Among the factors that could contribute to the pathogenesis of asthma in obese children there are mechanical, inflammatory, genetic, hormonal and immune factors (Figure 1).

Obesity may contribute to the pathogenesis of asthma due to mechanical (changes in airway mechanics), inflammatory (atopic VS non atopic inflammation, cytokine imbalance), genetic (genomic profile differences), metabolic (insulin resistance and decreased response to inhaled steroids) and immune factors (microbiome). It seems that physical inactivity and systemic steroid therapy could have a role in increasing the risk of developing obesity in asthmatic children.

### 3.1. Mechanical Factors

Obese patients show greater abdominal tissue deposition with subsequent increased abdominal pressure [17,33,34]. This abnormal tissue deposition is responsible for reduced chest expansion and consequent decrement in tidal volume and residual capacity. It seems that these mechanical changes could contribute to bronchial re-modelling over time, increasing airway obstruction and hyper-responsiveness and, therefore, the risk of asthma. 

Another factor playing a role in airflow reduction in obese asthmatics is dysanapsis [35,36]. Dysanapsis is a condition characterized by unequal growth of lung parenchyma and airway caliber and it has been observed in overweight and obese children with or without asthma [35,36,37]. This phenomenon would lead to higher FEV1 (forced expiratory volume in the 1st second), but even higher FVC (forced vital capacity), with subsequent reduction of FEV1/FVC ratio and airflow obstruction [35,37]. Forno et al. [35] examined data from six cohorts of children, aged 6–20 years, and evidenced that the dysanapsis observed in obese subjects resulted in an obstructive deficit, suggesting that the airflow obstruction seen in obese children is anatomical and/or developmental and not completely related to bronchospasm or airway inflammation. Furthermore, the authors demonstrated that in obese asthmatic children, dysanapsis has a clinical impact and may be partly responsible of asthma symptoms, reliever medication use, diminished response to ICS and severe exacerbations [35].

Interestingly, recent evidence [38] showed that obesity may increase susceptibility to air pollution in asthmatic children. It is known that air pollution, and in particular fine particulate matter (PM2.5), is dangerous for respiratory health since it promotes chronic bronchitis, incident asthma and asthma exacerbations [39]. Afshar-Mohajer et al. [38] evidenced that obese asthmatic children breathed at higher tidal volumes and had higher minute ventilation than normal weight children, resulting in more efficient fractional deposition of fine particulate matter in the lung. In particular, the authors found that an obese child (at the 99th percentile for BMI) had almost a 30% higher rate of alveolar PM2.5 deposition compared to a normal weight child (at the 50th for BMI). These findings suggest that obese children with asthma, in consideration of mechanical factors, were more vulnerable to air pollution and, subsequently, were at higher risk of more severe and uncontrolled asthma.

There is evidence that Mediterranean diet rich in fruit, vegetable and n-3 polyunsaturated fatty acids is protective for asthma development, exacerbations and asthma symptoms due to the positive impacts on inflammation, oxidation and microbial composition. Conversely, the Western diet—rich in saturated fatty acids and low in antioxidants—stimulates inflammation and may increase the risk of preschool wheezing and asthma with a negative effect on lung function [40]. In addition, childhood asthma may be influenced by maternal diet during pregnancy, particularly by the intake of certain foods such as fish or fruits and vegetables, and nutrients such as vitamin E, vitamin D, zinc or polyunsaturated fatty acids). In murine models, maternal high-fat diet increased airway hyperreactivity in offspring, and was associated with higher neutrophil percentage, greater total protein and higher IL-6 levels in bronchoalveolar lavage suggesting a role of maternal diet in programming adult offspring airway hyperreactivity [41].

However, a recent large study cohort on more than 18,000 mother-child pairs from seven European birth cohorts failed to demonstrate association between a pro-inflammatory and low-quality maternal diet during pregnancy and asthma or wheezing development in offspring [42]. Similarly, in a large prospective study gestational diabetes and gestational hypertension were initially associated with increased odds of overweight or obesity throughout childhood but this association became not significant when adjusted for maternal BMI [43]. Other maternal factors that may affect the risk of respiratory disease in offspring are pre-pregnancy overweight and obesity, and excessive gestational weight gain. The effect of maternal overweight/obesity on wheezing in offspring is more pronounced in the first years of life, while the effect on wheezing at school age is likely mediated by overweight and obesity in children [44]. 

### 3.2. Proinflammatory Factors

Proinflammatory factors driven by obesity may play a role in the pathogenesis of asthma [33,34]. Adipose tissue is an important source of pro-inflammatory cytokines and adipokines, particularly leptin. Fat accumulation and excessive BMI have been associated with hypoxia, focal adipocyte necrosis, and subsequent recruitment of macrophages [45]. This can lead to higher blood levels of leptin and other pro-inflammatory hormones and decreased levels of adiponectin, an anti-inflammatory adipokine [17,45,46]. Leptin, whom production increases proportionally with adipose tissue, has proinflammatory actions and promotes neutrophil chemotaxis, reactive oxygen species production, natural killer cells and macrophage activation and phagocytosis. Furthermore, leptin is responsible of T helper (Th)1 cytokines (IL-6 and IFN-γ) release and suppression of Th2 cytokines (IL-4) production [45,47]. Leptin receptors are expressed in human airway cells [45], and it has been hypothesized that leptin may be related to airway reactivity [33,48]. However, the precise mechanisms by which these inflammatory mediators contribute to inflammation in asthmatic airways are not well known as of yet [31,45]. In a study of 345 Saudi children, obese asthmatics had higher levels of leptin compared to nonobese asthmatics and leptin showed a weak correlation with IL-4, IL-5, and IL-21 [49]. Interestingly, children with a poor control of asthma showed significantly higher leptin levels than controlled asthmatic children suggesting a role of leptin as a potential predictor for asthma control as previously hypothesized by other authors [50]. 

Guler et al. observed that serum leptin levels of asthmatic children, in particular atopic boys, were higher than in healthy children [48]. A recent trial on 119 pregnant obese women demonstrated that offspring with high cord blood leptin have 30% higher asthma risk at age 3 [51].

Instead, adiponectin would have a protective role against asthma. Adiponectin is an insulin sensitizing hormone with anti-inflammatory function; indeed, it inhibits effects of proinflammatory cytokines (i.e., TNF-α, IL-6) and promotes expression of anti-inflammatory cytokines (IL-10 and IL-1 receptor antagonist) [45]. Adiponectin binding proteins are expressed in bronchial epithelium, airway smooth muscle and pulmonary vasculature [45]. Yuksel et al. [52] evaluated the serum levels of adiponectin in obese and non-obese children with asthma and in healthy children and found that adiponectin levels were decreased in obese asthmatic children, suggesting a correlation between decreased adiponectin levels, seen in overweight/obese subjects, and a greater risk of developing asthma. In murine models, adiponectin was found to be decreased by IL4, one of the cytokines associated with Th2 response, which instead stimulated lipogenesis and inhibited lipolysis [53].

### 3.3. Genetic Factors

There is increasing evidence that asthma and obesity have several genetic determinants in common [17,34]. One of the most important studies that evidenced possible shared genetic causes in asthma and obesity was performed in 2007 by Hallstrand et al. [54]. The authors analyzed 1001 monozygotic and 383 dizygotic same-sex twin pairs registered in the University of Washington Twin Registry using a structural equation modelling. They found that 8% of the genetic component of obesity was shared with asthma, suggesting a significant genetic pleiotropy between the two disorders. 

Two genes seem to be strongly associated with both obesity and asthma phenotypes, namely, β2 adrenergic receptor and tumor necrosis factor α [34,55]. Another interesting and recent study [56] confirmed the existence of a positive genetic correlation between obesity and asthma subtypes in adults. A genetic shared etiology of obesity-related traits with later-onset and non-atopic asthma was identified, while no genetic link was found between obesity and early onset and atopic asthma.

Alongside genetics epigenetics seem to be involved in determining the association between asthma and obesity [17,57]. Rastogi et al. [57] observed that the epigenetic footprint in obese asthmatic children was different from that observed in normal weight asthmatics, obese non-asthmatics and healthy children.

### 3.4. Hormonal Factors

Karampatakis and colleagues [58] proposed that the mechanism underlying the link between obesity and asthma relies on insulin resistance and glucose intolerance. The authors enrolled 71 pre-pubertal children, with and without asthma and with different body mass indices, and observed that obese asthmatic children with confirmed insulin resistance had higher bronchial hyperresponsiveness compared to obese children with normal glucose tolerance. Therefore, it was hypothesized that airway hyperreactivity in obese children is caused by insulin resistance and impaired glucose tolerance and not obesity alone. Insulin resistance seems to promote the development of Th1 inflammation through the production of pro-inflammatory molecules (i.e., IL-6, TNF-α) [17,59]. Furthermore, insulin resistance determines hyperinsulinemia, which is responsible for inhibiting pre-synaptic M2 muscarinic receptors, leading to bronchial hyperreactivity [17,60].

### 3.5. Microbiome

Another interesting hypothesis identifies gut microbiota alterations as playing an important role in the development of many diseases, including asthma and obesity [17,61]. It is thought that alterations to the microbiota in early life plays an important role in the development of asthma and obesity [31]. Some early life events, such as infant diet, delivery modality and early exposure to antibiotics may lead to microbiome alterations and long-term consequences, including an increased risk of asthma, diabetes and obesity [31,62,63]. Bokulich et al. [63] enrolled 43 US infants and followed them to the age of 2 years, collecting stool samples and studying delivery modality, feeding and systemic antibiotic exposures. The authors evidenced that a caesarean section depleted Bacteroidetes populations, altering the establishment of maternal bacteria, possibly because of a decreased exposure to maternal microbes during birth. Formula feeding altered microbiome diversity and decreased microbiota maturations during the first 1–2 years of life; furthermore, early life antibiotic exposure delayed microbiome development and suppressed *Clostridiales*. 

Alterations in the gut microbiome could be determined also by some dietary patterns, in particular the Western one, high in fat and low in fibers [64]. A low fiber diet was associated with alterations in gut microbiome and decreased levels of short chain fatty acids (SCFAs), metabolites of fibers. SCFAs are thought to protect against allergic inflammation whereas a low-fiber diet decreasing their levels may increase allergic airway disease.

The mechanisms underlying the increased risk of obesity due to asthma are unclear. It has been hypothesized that physical inactivity and steroid therapy may have a role.

### 3.6. Physical Activity

It has been proposed that asthmatic children are at a greater risk of becoming overweight and obese than non-asthmatic children, because they tend to be less physically active, for fear of asthma exacerbation [30,65]. The factors that affect participation in physical activity in children and young people with asthma could be influenced by family illness beliefs and behaviors, organizational policies (i.e., school), health care advice, and, overall, an incorrect perception and attribution of asthmatic symptoms [65]. 

Souza de Almeida et al. [66] conducted a cross-sectional study on 156 young asthmatics in order to study the correlation between obesity and the risk and severity of exercise-induced bronchospasm in asthmatic children and adolescents. They observed a greater risk of exercise-induced bronchospasm in obese individuals with asthma, compared to non-obese peers. The authors suggested that exercise-induced bronchospasm, rising respiratory symptoms in obese asthmatics, would determine a lower participation in physical activities, sedentarism and lower cardiovascular conditioning, resulting in a vicious circle with further weight gain and worsening of asthma control.

### 3.7. Steroid Therapy

Another factor that may contribute to the development of obesity in asthmatic children is the potential adverse effects of systemic glucocorticosteroid therapy [30,32,67]. A long-term systemic treatment with glucocorticosteroids could promote lipid deposition in tissues, especially in the shoulders and in the trunk [30,67]. It is important to underline that the effects of systemic glucocorticosteroids depend on both the dosage and the duration of the treatment [67]. 

Interestingly, Chen et al. [30] discovered a preventive role of rescue medications in developing overweight or obesity in asthmatic children. The authors, after enrolling more than 2000 children, observed, during a 10-year follow-up, that children who used rescue asthma medications showed a decreased risk of developing asthma. They suggested that beta-agonists could have effects on adipocytes, where beta2 adrenergic receptors are present, and promote lipolysis, with a subsequent protection against obesity.

## 4. The “Obese-Asthma” Phenotype

Clinical manifestations of asthma are highly heterogeneous. Molecular pathways implicated in asthma pathogenesis are different among selected subjects. Traditionally, classic childhood asthma has a Th2 phenotype with eosinophilic inflammation and history of atopy [68]. Interestingly, severe asthma in children has been associated with atopy, bronchial eosinophilic inflammation and multiple aero-allergen sensitization [69]. 

Asthma symptoms can increase in terms of prevalence and severity when children living with asthma also have obesity, and epidemiological evidence exists indicating that asthma that develops as a consequence of obesity is not of an allergic type [70]. The existence of an “obese asthma” phenotype, in which high body weight changed asthma characteristics, has also been reported [71] (Table 2).

This phenotype has a Th2 low profile with predominant neutrophil infiltration in the bronchial mucosa as well as low IgE and low eosinophilic infiltration [72,73]. The low-grade systemic inflammation state of obesity chronic low-grade inflammatory condition, called “meta-inflammation” [74], is sustained by leptin and by the relative hypoxia promoted by the proliferation of adipocytes and their subsequent death [75]. Following adipocyte death, macrophages M1 (stimulators of pro-inflammatory factors and inducers of insulin resistance) produce inflammatory cytokines such as IL-6, TNF-α, IL-1β, and monocyte chemoattractant protein (MCP-1). MCP-1 promotes the recruitment of monocytes and their differentiation into macrophages [76]. Macrophages activate Th1 and Th17 cells with subsequent neutrophilic inflammation [77], and the release of IL-17, IL-21, and IL-IL-17 is responsible for the recruitment of neutrophils and is associated with airway hyperreactivity (AHR), severe asthma and corticosteroid resistance [17,78,79,80,81]. Type-3 innate lymphoid cells (ILC3) secreting IL-17 also appear to play a key role in neutrophilic corticosteroid-resistant asthma [82]. Furthermore, Th1 polarization correlates with leptin and the production of IL-6, which further activates neutrophils [68]. IL-6 is a pro-inflammatory cytokine excreted from T cells, myeloid lineage cells, and endothelial cells whose receptor IL-6R is expressed by neutrophils [83]. Neutrophils are the main source of IL-6 generated in the airways of asthmatic patients [84] and increased levels of IL-6 have been found in the sputum, serum, and bronchoalveolar fluid of asthmatic patients, especially in those with severe asthma. Furthermore, IL-6 has been found associated with severe asthma in adults [85,86] and in obese subjects [87]. The potential bridging role of IL-6 between asthma and obesity has been sustained by the increasing levels of IL-6 with BMI in children, and by the correlation between IL-6 with and the probability of asthma exacerbation [88]. 

In adults with asthma, an elevated level of serum IL-6 was associated with increased body weight, lower lung function and greater exacerbation risk [85]. The inflammatory response evidences a Th1 polarization and also the expression of high levels of interferon (IFN) –gamma which promotes AHR [89]). Table 3 reports some of the studies on the underlying mechanisms of the neutrophilic inflammation of the Th2-low endotype implied in the “obese asthma” phenotype. Rastogi et al. found evidence of non-atopic systemic inflammation, with monocyte activation and Th1 polarization, among obese asthmatic adolescents. This systemic inflammation is a potential mechanism linking metabolic dysregulation and pulmonary function deficits among the obese patients [90]. In the Severe Asthma Research Program (SARP), two main “obese asthma” phenotypes were examined “early onset obese-asthma” and “late-onset obese-asthma”.

Holguin et al. described an early onset phenotype including patients <12 years of age. Patients belonging to this group necessitated a greater use of health care resources and suffered from a diminished quality of life, higher airway obstruction with marginally higher maximal FEV1 reversal and increased bronchial hyperresponsiveness. They also evidenced high IgE levels and positivity for skin prick test (T2 high).

The late-onset phenotype identified obese patients >12 years of age, who were older at the time of diagnosis, often female, with a higher frequency of severe asthma and a poorer disease control. This late-onset phenotype had a low Th2 profile with predominant neutrophil infiltration as well as low IgE and low eosinophilic airway infiltration [91].

Chen et al. investigated the effects of asthma and related phenotypes on the development of obesity in a cohort of non-obese children. They examined the incidence of obesity over a 10-year follow-up to assess the hypothesis that children presenting with asthma in early life are at increased risk of developing obesity during childhood and adolescence. In particular, non-obese children with asthma at baseline were 51% more likely to develop obesity during follow-up compared with children without asthma at baseline.

Moreover, patients who used asthma rescue drugs at study entry had a significantly lower risk of becoming obese during the follow-up compared with participants who did not use asthma therapies. 

Their results suggested that using asthma rescue medication in early childhood might protect against weight gain in later life. In conclusion, Chen and colleagues identified potential to prevent obesity through the early diagnosis and treatment of childhood asthma in asthma therapies the [30]. Table 3 shows studies reporting the underlying mechanisms of the Th2-low endotype implied in the “obese asthma” phenotype.

**Table 3:** Studies reporting the underlying mechanisms of the Th2-low endotype implied in the “obese asthma” phenotype.

Articles	Subjects	Description
Liang L 2018 KJIM [92]	Murine models	Mice on high fat diet showed allergic airway inflammation. Blockading of IL-17 decreased airway hyper-responsiveness (AHR) and airway inflammation. The administration of the anti-IL-17 antibody decreased the leptin/adiponectin ratio, inhibited airway inflammation and AHR, and increased adipokine levels.
Scott HA 2011, ERJ [93]	Obese (*n* = 68) and nonobese (*n* = 47) adults with asthma, and obese (*n* = 16) and nonobese (*n* = 63) healthy controls	Sputum neutrophil percentage was positively associated with BMI in females with asthma and neutrophilic asthma was present in a greater proportion of obese compared with non-obese females.
Telenga ED 2012 [94]	276 asthmatic patients (53 bese)	Obese women had significantly higher blood neutrophils. After a two-week treatment with corticosteroids, less corticosteroid-induced improvement in FEV-1% predicted was observed in obese patients than in lean patients.
Kim HY 2014 Nat Med [95]	Murine model	In obese mice airway hypereactivity (AHR) was dependent on IL-AHR was also associated with the expansion of type 3 innate lymphoid cells producing IL-17.

## 5. Treatment of the Obese-Asthma Phenotype

The mechanisms that explain the pathophysiology of obesity related non-Th2 asthma are complicated. It is evident that the current approach for childhood asthma management may not be effective in obese children with asthma. Indeed, obese children with asthma may be poorly reactive or non-reactive to currently available drugs for asthma control [96].

Steroids are less effective in obese than in lean individuals with asthma [97]. Compared to subjects with normal BMI, obese subjects, particularly those with marked obesity, had a reduced chance of achieving asthma control. Part of this reduced efficacy of corticosteroids in obese asthmatics may be related to obesity-related changes in the anti-inflammatory effects of these agents. The ability of dexamethasone to induce MKP-1, a glucocorticoid responsive gene, in peripheral blood mononuclear cells (PBMCs) and in bronchoalveolar lavage (BAL) cells is reduced in obese versus lean asthmatics [98]. In particular, studies indicated that obesity reduces the response to steroids and that there might be an additional important reason as to why obese asthmatics do not respond well to these agents: steroids target the immune processes that mediate allergic responses, but many obese subjects with severe asthma are non-atopic [91,99]. 

Orries et al. [29] analyzed data from 566 children aged 4–13 years with asthma under ICS as maintenance therapy and showed that obese children with moderate to severe asthma had less adherence to therapy than those with mild asthma. The increased risk of non-adherent behavior reported by parents in children with excess weight appeared to be driven by intentional non-adherence, which was particularly evident in children aged from 4 to 10 years. A correlation between excess weight and general non-adherence to ICS has been demonstrated mainly in children with moderate to severe asthma who exhibited intentional non-adherence behavior. Their findings suggested that a more careful monitoring of children with excess weight and asthma is necessary. 

### 5.1. Exercise, Weight Loss, Diet

Non-atopic systemic inflammatory patterns of the “obese asthma” phenotype are connected with lower airway obstruction and exercise-induced bronchoconstriction [100]. One of the first ways to treat obesity-related asthma is to lose body weight. Some studies have demonstrated that weight loss improved asthma disease, particularly in relation to the pulmonary function, although there was no modification in systemic inflammation [101]. 

The first line treatment for paediatric obesity is family-based intervention that involves a combination of lifestyle approaches including moderate energy intake, increase physical activity, reduced sedentary activities and promotes the collaboration of the whole family to modify current family dietary and activity patterns.

Weight reduction in asthmatic children can lead to a better prognosis of asthma through a better quality of life related to asthma, increased asthma pulmonary control and better lung function. It has been reported that even a 5–10% reduction in weight can lead to improved asthma outcomes [102].

In their study, Willeboardse et al. [103] recruited 87 asthmatic overweight or obese children aged 6–16 years old. Children received long-lasting multifactorial intervention (18 months), consisting of sport sessions, parental involvement, individual counselling and lifestyle advice, including dietary advice and cognitive behavioural therapy. The control group received the usual care. The authors demonstrated that clinically relevant improvements in body weight, lung function and asthma characteristics occurred in both the intervention and control groups, although some effects were more pronounced in the intervention group such as FVC, asthma control and quality of life. Weight reduction intervention can be clinically beneficial for children with asthma.

Clarke et al. published a qualitative study exploring weight management in families living with pediatric asthma. The authors pointed out that families felt unsupported and ill-equipped to manage the weight of obese asthmatic children and expressed their uncertainty about how to maintain control of asthma through health-promoting behaviours. Families stressed the advantage of cooperation with health professionals to raise their concerns and the possibility of creating individualized management plans to follow in a comfortable and safe environment [104].

Intense physical activity plays a critical role in adequate growth and development, and is a useful therapy for chronic diseases [105]. 

In a systematic review, Leinaar and colleagues analyzed the relationship between asthma and overweight or obesity in youth, considering the role of physical activity as a mediator in this relationship. They found that physical activity predisposed asthmatic children to decreased physical exercise with subsequent weight gain. Several possible determinants could be implicated in the association between asthma and physical activity in children, such as symptoms of asthma or exercise-induced asthma, negative self-perception of physical ability, and parental perceptions of risk associated with physical exercise in asthmatic youth [106].

Lu et al. studied a cohort of 665 children (6–11 years old; 49% males) and explored the relationship between asthma outcomes and aerobic fitness (measured by endurance time), self-reported sedentary time and BMI categories. They suggested that aerobic fitness and sedentary time should be included in assessments and the management of asthma in children. They also found that increased sedentary time was associated with worse asthma outcomes [107].

Onur et al. recruited 13 control and 30 asthmatic children. Their study results revealed that the use of constructed exercise programs in asthmatic children ameliorated lung function. The underlying mechanism may be an increase in oxidant capacity, resulting in a decreased oxidative burden, together strengthening the anti-inflammatory effects of steroids. Exercise may operate synergistically with inhaled steroids to valorize lung function [108].

In a cross-sectional study, 122 school-aged children, who were divided into four groups (healthy control, asthma, overweight/obesity and asthma, overweight/obesity), performed a pulmonary function test wearing an activity monitor for 7 days. In this study, Willeboordse et al. pointed out that the exercise levels were low in the entire study population, leading to increased health risks. Unlike previous studies, the authors found no significant associations between asthma, overweight and exercise levels in school-aged children compared to peers without asthma and/or overweight or obese. In light of these conflicting data, the authors also pointed out the need to investigate the role of modifiable risk factors, such as physical activity and diet, in the management of children with the difficult-to-treat phenotype “obese asthma” [109].

Epidemiologic studies suggested that specific dietary features provide both a decreased risk of asthma and the enhanced management of existing asthma [110]. Trompette et al. supported the concept that diet-focused intervention strategies are a valuable approach not only for bowel disease but also for respiratory inflammation. In fact, they demonstrated that the content of dietary fiber influences the intestinal microbiota and therefore the levels of the concentration of circulating short-chain fatty acids. SCFAs, such as propionate, enhance the hematopoiesis of dendritic cell precursors from the bone marrow and these cells show a reduced ability to activate Th2 effector cells in the lung. As a result, allergic airway inflammation cannot be sustained and resolves quickly. This work highlights the importance of fermentable dietary fibers and provides a cellular mechanism for an intestinal marrow-lung-bone axis in the control of allergic airway inflammation [111].

In a randomized controlled pilot study, with obese asthmatic children aged 8 to 17 years, Jensen and colleagues found that dietary intervention induced acute weight loss with subsequent improvements in static lung function and asthma control, while systemic and airway inflammation did not change after weight loss. The authors emphasized the role of diet-induced weight loss to achieve significant improvements in clinical outcomes for obese children with asthma [101]. 

Several studies have demonstrated that fish, omega-3 fatty acids, fresh fruits, vegetables and low saturated fat content food, might be associated with reduced risk of asthma development and better control of existing asthma [112].

In particular, Lang et al. demonstrated that an adequate intake of omega-3 fatty acids may improve asthma control in obese asthmatic patients through several anti-inflammatory mechanisms [113]. 

In addition, several analyses found that pre-treatments with omega-3 fatty acid supplements, prior to physical activity, might lead to reduced asthma symptoms [114].

However, the most effective intervention for producing significant weight loss is bariatric surgery. Bariatric surgery has been reported to promote highly significant developments in asthma control, airway reactivity and lung function in all studies [115]. Tt was also found to have significant success in terms of asthma exacerbations. Hasegawa et al. demonstrated that bariatric surgery led to a nearly 60% reduction in the risk of having an asthma exacerbation, with a baseline risk of asthma exacerbation in the population of approximately 22% [116].

### 5.2. Pharmacotherapy

No specific medications are recommended for obesity in paediatric patients with asthma, as children are treated according to the frequency and severity of symptoms and their impact on daily activities. Weight loss drugs are recommended only for children with severe obesity-related complications [117].

Orlistat is the only widely available and authorized medication for weight loss in children and little is known about its effects on asthma. Metformin is another drug that requests additional investigation in the perspective of obese children with asthma; it is usually suggested to obese adolescents with type 2 diabetes. The fact that metformin supports weight loss and may have an appropriate action to lung health, makes it an attractive target for further investigation [118].

### 5.3. Vitamin D

There is growing literature on the potential relationship between vitamin D deficiency, defined as 25-OH vitamin D < 20 ng/mL [119], and the development of obesity and asthma besides a higher risk of respiratory infections and decreased corticosteroid responsiveness asthma [63,68,120]. Lautenbacher and colleagues [121] found that obese asthmatic children, affected by vitamin D deficiency, had lower FEV1, TLC (Total Lung Capacity) and FRC (Functional Residual Capacity) compared to obese children with normal vitamin D levels. These findings suggested that vitamin D deficiency could play a role in determining lower airway obstruction and reduced pulmonary volumes among obese asthmatic children, even if the exact underlying mechanisms are poorly understood [121]. 

Considering that vitamin D deficiency was associated with a lower pulmonary function in obese children, vitamin D supplementation has been proposed to improve asthma severity and control [47,68,122,123,124].

Tachimoto et al. [122,124] conducted a trial on asthmatic children, studying the effect of vitamin D supplementation on asthma control. Asthmatic children who received vitamin D supplementation for two months showed a better asthma control and fewer exacerbations if compared to children who did not take vitamin D supplementation. 

The exact mechanisms by which vitamin D supplementation influences asthma control in obese children are not understood, but it is known that vitamin D leads to immunomodulatory effects, and it could influence intestinal microflora, a mechanism involved in asthma pathophysiology [47]. Furthermore, vitamin D has anti-inflammatory properties, and, for this reason, it could play a role in controlling non-atopic asthma [123]. The optimal dose of vitamin D in supplementation for asthma is still a subject of study [123]. Even if there is a growing interest in the potential role of vitamin D supplementation in improving asthma management, there is not enough evidence to recommend its use in all asthmatic children [122,123].

## 6. Management of “Obese-Asthma” Comorbidities and Potential Triggers

Because of the complexity of the conditions involved, obesity-associated asthma is understood to be a multifaceted condition requiring a multidisciplinary approach. Besides the treatment, various comorbidities associated with obesity and triggers have to be considered, since both can eventually complicate asthma control [125].

Gastroesophageal reflux disease (GERD) is very common in severe asthma [126,127], especially in obese patients. In the Severe Asthma Research Program (SARP), subjects in the obese asthma clusters more frequent experienced GERD than subjects in the other severe asthma clusters [128]. GERD might exercise a deleterious effect by inducing vagal reflex, neuroinflammation and directly triggering airway inflammation. In a recent study, obese patients with severe asthma and GERD showed epithelial dysfunction. This is caused by an altered expression of chemokines and a dysregulated inflow of intracellular calcium into the epithelial cells of the airways [129].

Several large, randomized placebo-controlled clinical trials have evaluated the ability of proton pump inhibitors to ameliorate asthma symptoms, but most identified only a modest effect or no effect of treatment, although none of the studies focused specifically on obese asthmatics [130].

In obese patients with severe asthma, there is also a high prevalence of obstructive sleep apnea (OSA) with rates as high as 80% [131]. Wang et al. demonstrated that the frequency of severe asthma exacerbations was higher in asthmatics with OSA [132]. OSA is associated with systemic inflammation, and in obese patients, the intermittent hypoxemia associated with repeated obstructive episodes may exacerbate already existing adipose tissue hypoxia, worsening adipocyte death, macrophage infiltration and consequent systemic inflammation [130]. There is evidence that continuous positive airway pressure (CPAP) may reduce systemic inflammation in obese asthmatics and also decrease airway responsiveness, asthma symptoms and SABA use [133]. Bariatric surgery improves both asthma and OSA [134].

Besides GERD and OSA, there are other conditions or comorbidities that may contribute to worsening respiratory symptoms in asthmatic patients: dysfunctional breathing, sedentary lifestyle and consequent deconditioning [135,136] and exposure to smoke [137].

Dysfunctional breathing is a respiratory disorder, characterized by an alteration in breathing patterns that cannot be referred to a specific medical diagnosis, such as asthma or chronic obstructive pulmonary disease. It is frequent and affects nearly one third of female and one fifth of male asthmatics [138]. Dysfunctional breathing could determine chronic or recurrent respiratory symptoms like dyspnea, exercise-induced breathlessness, deep sighing, frequent yawning and hyperventilation [135,139]. For this reason, it could be responsible of overestimation of the severity of respiratory symptoms in asthmatic patients, leading to a possible overtreatment [135]. Additionally, a sedentary lifestyle and consequent cardiovascular deconditioning could play a role in the development of respiratory symptoms, especially during exercise [140], in obese asthmatic patients [136]. Shim et al. [140] demonstrated that the most frequent cause of breathlessness in obese adolescents, with or without asthma, was cardiopulmonary deconditioning. As a result, it is important to promote physical activity in asthmatics in order to interrupt a possible vicious circle of physical inactivity, deconditioning and respiratory symptoms [141].

Smoke exposure is another important risk factor for all asthmatic children and adolescents. Both passive and active smoking is associated with steroid resistance in children with asthma [142]. In a large national study conducted in Scotland, Mackay et al. described the dramatic impact of secondhand smoke exposure on asthmatic children, and they showed a significant reduction in asthma flare-ups after the introduction of a smoking ban in public places. These data suggested that the reduction of exposure to secondhand smoke leads to a better control of asthma in children [143]. Kitsantas et al. in a cross-sectional study showed that obese adolescents are more likely to be diagnosed with asthma when exposed to ambient tobacco smoke inside the home [144].

Furthermore, Wu et al. compared two observational studies in cohorts of urban children with asthma and they showed that exposure to secondhand smoke was associated with the worsening of symptoms in overweight /obese children compared to normal weight children, suggesting that a high body mass index may increase susceptibility to secondhand smoke. These findings offer new insights into why asthma in overweight and obese children may be more severe [137]. 

Second-hand smoke exposure has a significant impact on both innate and adaptive immunity, and this may be a potential mechanism by which this factor may affect and/or promote “obese asthma”, alongside recurrent respiratory infections. The precise mechanisms through which cigarette smoke interfere with the immune system are not completely clear. Marseglia et al. [145] conducted a study that included 128 children who performed adenoidectomy; they studied the adenoid tissue that was surgically removed, and they evidenced that children exposed to passive smoke had less Th1 adenoidal lymphocytes (IFN-γ–CD8+) than children not exposed to passive smoke. The reduction of Th1 adenoidal lymphocytes, resulting in reduced IFN-γ levels, is responsible for the decreased production of chemokines and adhesion molecules and, therefore, leads to a defective immune response with subsequent important recruitment of host defence cells [145,146]. Furthermore, second-hand smoke can affect the production of several cytokines at different levels in the respiratory tract, which overall may impact on asthma in the context of united airways approach, passing through an altered responsiveness to infectious agents as well [145,146,147,148]. Gentile et al. [146]) evidenced that children exposed to tobacco smoke had persistent diminished dendritic cell IL-10 production during infancy and suggested that this may be the mechanism by which exposure to tobacco smoke promotes the development of asthma. Hossny et al. [148] demonstrated that low serum IL-18 levels played a role in promoting exacerbations in asthmatic children but did not detect an association between passive smoke exposure and IL-18 serum levels. Further studies are needed to precisely understand the immunological background of tobacco smoke exposure in the pathogenesis of asthma.

Therefore, smoke exposure should be identified and eliminated immediately after a diagnosis of asthma, before starting pharmacological treatment; the family should be assisted in finding supportive strategies, along with pharmacotherapy, to quit smoking and limit exposure to this risk factor [149].

Healthcare professionals should always try to identify potential environmental risk factors as they can represent serious asthma triggers, especially among adolescents with obesity.

**Figure 2:**
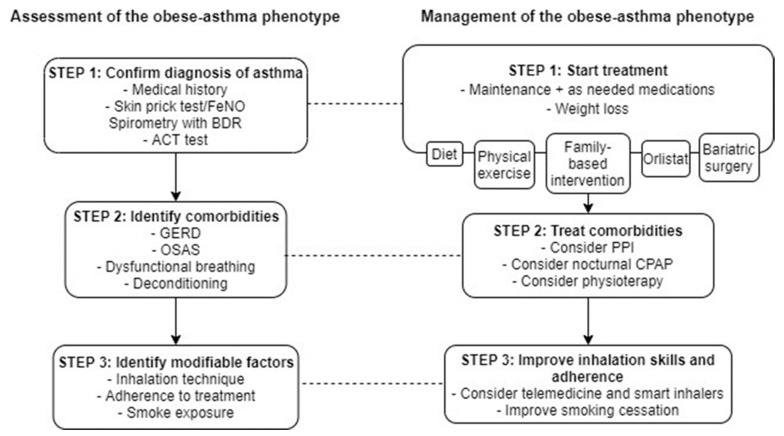
Step-wise assessment and management of the obese-asthma phenotype. FeNO: Fractional exhaled Nitric Oxid. BDR: Bronchodilator Response. ACT: Asthma Control Test. GERD: Gastro-Esophageal Reflux Disease. OSAS: Obstructive Sleep Apnea Sydrome. PPI: Protonic Pump Inhibitor. CPAP: Continuous Positive Airway Pressure.

In Figure 2, we proposed a step-wise assessment and management of the child with the obese-asthma phenotype. In eosinophilic asthma, confirm the diagnosis of asthma and assessing the comorbidities and modifiable factors are essential steps. This approach is particularly important in problematic asthma [150]. Once the basic elements of asthma are assessed, management includes starting pharmacologic and non-pharmacologic treatment to treat both asthma and the associated comorbidities, and to identify the modifiable factors responsible for poor control.

## 7. Future Prospective

A recent increase in both asthma and obesity in young people has led to increased health care utilization. It is important to provide high-risk children and adolescents with strategies to promote physical exercise and healthy habits and enhance asthma self-management and treatment adherence to decrease long-term morbidity and mortality.

The development of hand-pocket tools, such as electronic monitoring devices and apps may facilitate communication with young patients with asthma and obesity. Reminders improve adherence to treatment, and apps or serious games can facilitate the understanding of the disease and promote actions to improve asthma control, thereby facilitating early intervention and eventually reducing healthcare costs. Lv et al. evidenced in the success of using a nurse-led mobile technology oriented approach to improve the management of asthma care among children. The authors established a reduction in asthma exacerbations and participants improved adherence to treatment [151]. Fedele et al. evaluated the use of mobile health technology that incorporates behavioral lifestyle interventions and nurse-facilitated self-management for families of children aged 6 to 12 years with asthma and obesity. This was the first study to develop nurse-delivered behavioral family lifestyle intervention (BFI), which was supported by an mHealth app and tailored to the needs of children with asthma and obesity with the aim of offering self-management skills of parents [152]. At present, BFIs are considered to be the gold standard for promoting effective weight self-management in children [153]. Nichols et al., in the MATADORS study, explored multi-morbid chronic conditions, in particular asthma and obesity, and associated symptoms, using mobile health technology to directly improve self-management skills and health outcomes of young people. This is considered to be a pilot study, that has not yet been conducted, investigating the use of mHealth technology in disease management for a targeted population, so as to improve symptom self-management and drug adherence to reduce future disease burden [154].

This paper provides a comprehensive review of the relationship between obesity and asthma in children and highlights the differences of the obese asthma phenotype with the allergic eosinophilic phenotype. Asthma and obesity are two important health problems in children and adolescents. Their prevalence has increased simultaneously in recent decades, leading to a greater number of studies that have shown the existence of a complex relationship between the two conditions. The identification of the phenotype obesity-related asthma is crucial to start specific therapeutic management since the underlying mechanisms differ from Th2 high eosinophilic asthma, and its management often requires a multidisciplinary approach.

## 8. Conclusions

The identification of biomarkers related to obese asthma represents an active area of research, aiming to distinguish the endotype of each patient and contribute to tailored management and treatment. For Th2 asthma, the pathogenesis has been extensively investigated leading to the development of targeted therapies such as biologics, but in the obese asthma phenotype specific therapies are lacking. Besides the cornerstones of asthma management (assessing and solving modifiable factors), in obese patients lifestyle should be optimized with interventions aiming to promote physical exercise and healthy diet, and associated comorbidities such as GERD or OSA have to be assessed. A step-wise approach involving different specialists can be crucial to obtain good control in obese patients with asthma. In children and adolescents, electronical devices and apps can motivate behavioural change, improve the self-management of symptoms and medication adherence, thereby decreasing the risk of exacerbations and therefore the burden of the disease. Prospective studies are required to further identify high-risk groups and develop effective interventions to modify risk behaviours that may contribute to both obesity and asthma. 

## Figures and Tables

**Table 1:** Studies supporting the higher risk of overweight and obese subjects of developing asthma.

Authors	Type of Study	Recruited Sample	Population	Main Outcomes
Davis A et al., 2007 [20]	Cross-sectional study	471.969 adolescents	Adolescents	Current and lifetime asthma prevalence increased as BMI percentile increased starting with the 25th to 35th percentile group and with the 45th to 55th percentile group, respectively.
Tsai HJ et al., 2018 [18]	Prospective study	1928 children (enrolled at birth and followed prospectively).	Mean age 7.8 ± 3.3 years	Excessive early life weight gain and overweight were both associated with an increased risk of asthma in childhood.
Contreras ZA et al., 2018 [19]	Analysis of 16 European cohorts	21,130 children	Mean age 4.1 ± 0.6 years	Early onset wheezing and asthma were associated with higher incidence of childhood obesity. Obese children have a double risk of developing asthma with a risk proportional to BMI values, particularly in boys.
Lang JE et al., 2019 [22]	Retrospective cohort study	507,496 children	Children and adolescents aged 2–17 years	Obesity increased asthma risk in all age groups but especially in the prepubertal school-aged group (7–11 years) without allergic rhinitis.
Barross LL et al., 2011 [23]	Cross-sectional study	508 subjects	Adults	There was a positive association between BMI and uncontrolled asthma. Between severe asthmatics, the obese had higher rate of hospitalization and emergency room accesses.
De Jesus JPV et al., 2018 [24]	Cross-sectional study	925 subjects	Adults	Compared to non-obese asthmatics, obese asthmatics have: • poorer asthma control • lower spirometric values • poorer quality of life • more frequent asthma exacerbation requiring oral corticosteroids

BMI, body mass index.

**Table 2:** The two phenotypes of childhood asthma.

	“Classic” Asthma Phenotype	“Obese Asthma” Phenotype
Prick tests for inhaled allergens	Positive	Negative
Biomarkers:		
- FeNO	High	Low
- Blood eosinophils	High	Low
- IgE levels	High	Low
Inflammation pattern	Th2 polarization	No Th2 polarization (Th1 or Th17 polarization)
Cells involved	Th2 lymphocytes, type 2 innate lymphoid cells, eosinophils, mast cells	Neutrophils, type 3 innate lymphoid cells, macrophages
Inflammatory cytokines	IL-4, IL-5, IL-13	IL-6, IL-17, IL-21, IL-22, IFN-gamma
Airway inflammation	Mainly eosinofilic	Mainly neutrofilic
Disease control/Response to steroid therapy	Generally good	Generally poor

FeNO: Fractional exhaled Nitric Oxid. IL: interleukin; IFN: interferon; Th: T helper.

## Data Availability

Not applicable.
